# Minutes of the Regular Monthly Meeting of the West Texas Medical Association

**Published:** 1899-07

**Authors:** Jno. S. Lankford, Wm. E. Luter

**Affiliations:** President; San Antonio, Texas; Secretary; San Antonio, Texas


### Society Notes.

### Minutes of the Regular Monthly Meeting of the West Texas Medical Association.

SAN ANTONIO, TEXAS, May 25, 1899.

The regular monthly meeting of the West Texas Medical Association was held in the Hicks building on May 25, 1899, at 8 o'clock p. m., with Dr. Jno. S. Langford in the chair and Dr. William E. Luter, secretary. Those present were Drs. Jno. S. Langford, J. H. Bindley, Jno. V. Spring, B. F. Kingsley, B. E. Hadra, F. Paschal, R. E. Moss, T. T. Jackson, H. D. Barnitz and Wm. E Luter. The visitors were Drs. McPherson Barnitz and R. H. Sommerville. The minutes of the previous meeting were read, and with slight corrections were approved. Dr. Paschal was granted further time in which to report as chairman of the committee on streets. Dr. Spring, as chairman of a committee appointed to investigate a resolution passed at our September, 1898, meeting, regarding a revision of the text-books now in use in our public schools, reported that the committee had neglected to investigate the resolution until now; the State Medical Association had met and adjourned and the Legislature will adjourn in the next few days, and consequently it is too late this year to do anything with the resolution. Dr. Spring said: "My reason for offering the resolution at the time was to try and keep our association in line with the progress of the times. It is a fact that other State medical associations are moving in this direction. They see the necessity of better text-books on hygiene, physiology, alcoholics and narcotics for our public schools. The Mississippi State Association has appointed a committee to act with the committees from the State Dental Association and State Superintendent of Public Instruction to lay the matter before the State Legislature and secure the necessary laws to make it compulsory to give special instruction in physiology, hygiene, etc., etc. The State Medical and Dental Associations of Alabama have also joined hands by appointing committees, and they, too, are working to have the necessary laws enacted to secure a thorough revision of the text-books used in the public schools of that State on the subjects above mentioned. In our own State, Texas, the State Dental Association has been making this fight alone, and I feel assured that any one who will take the trouble to examine our public school text-books will find them not only seriously defective as to the subjects treated, but in many instances grossly erroneous as to facts. See, Hutchinson's Physiology and Hygiene, Human Body, by Martin, and Steele's Sciences. My original resolution was offered with the hope that we should endorse this movement and co-operate with our State Dental Association in their effort to secure the necessary legislation."

Dr. B. E. Hadra mentioned that members of the Austin Medical Association had suggested the uniting of the two associations—the Austin with the West Texas Medical Association, quarterly meetings to be held alternately in San Antonio and Austin, continuing at the same time our regular monthly meetings.

Upon motion, Drs. Hadra, Spring and Kingsley were appointed a committee of three to confer with the Austin Medical Association, and discuss the matter.

The report from the board of censors, recommending for membership Drs. R. H. Sommerville, McPherson Barnitz and J. D. Forest, was read, and upon motion, adopted. And the above three gentlemen were voted upon, respectively, and unanimously elected to active membership of this association.

The following communication was read:

"The DeWitt County Medical Association will entertain the South Texas Medical Association in Cuero, June 13 and 14, 1899. Members of the West Texas Medical Association are cordially invited to be present.

"Dr. J. H. Burleson,
(Signed) "Dr. J. M. Lackey,
"Dr. Walter Shropshire,
"Invitation Committee."

Upon motion, the secretary was instructed to accept the invitation, and advise that all members who could do so would attend.

Dr. Frank Paschal reported an exceedingly interesting case of carbolic acid poisoning: Patient, female; 23 years old, who took an ounce of pure carbolic acid with suicidal intent. She was given four ounces of sweet oil ten minutes afterward, and one half hour later she was in collapse, and was given one-fortieth grain strychnia hypodermically, stomach washed out with soap-suds and then put six ounces of oil in the stomach, one-twentieth of a grain of strychnia an hour later, and repeated in two hours. Four hours later she vomited freely, and the bowels were well moved. Digitalis fomentations were applied over the kidneys, vomiting and purging and restless, and pain over the kidneys, and passing of small amounts of urine. One-fortieth of a grain of strychnia hypodermically was given every four hours, also one-fourth of a grain of morphia every four hours, as required to relieve pain. She was nourished by enemas for two days, then was given small amounts of nourishment by the stomach, and allowed all of the water she cared to drink. The urine contained a small amount of albumen, but no blood. The patient recovered.

Dr. Kingsley stated that the case showed that no rules could be laid down for treatment, and the result in this case proved that the treatment was well carried out.

Dr. Hadra alluded to the various degrees of susceptibility, and referred to one case in which he gave two drops in intermittent fever, and the man complained that he felt as though poisoned. Also alluded to a baby who swallowed one drachm and got well. Dr. Hadra asked the question whether it acted as a reflex from the stomach or is absorbed and acts on the brain.

Dr. Jackson spoke of two cases of carbolic acid poisoning, and both died. One died in one-half hour and the other in two or three minutes, which he said showed that it acts reflexly.

Dr. Paschal said that if he were called upon to treat another case he would give strychnia in larger doses, from one-tenth to one-fifth of a grain.

Dr. Lankford mentioned a case in which he injected one-half pint solution of creolin (creolin one drachm to one-half ounce of water) into the rectum, and produced alarming symptoms, which however passed away.

Dr. Hadra spoke of the use of carbolic acid in tetanus, and said he had tried it, but with no more success than he had met with in the use of other remedies. He referred to an article by H. C. Wood, in *Merck's Archives* for May, 1899, on the use of carbolic acid in tetanus, where it was reported that out of thirty-four cases of well marked tetanus only one death occurred, perhaps due to too small doses. This, he thought, was wonderful, and out of the great number of cases of traumatic tetanus, which he had seen during his practice, he had never seen one get well, and he doubted the diagnosis of those cases where recovery was reported. He has used carbolic acid in epilepsy, and with wonderful effect. He thinks it acts as an antitoxin to the toxin of epilepsy. He begins with two drops three times a day, and gradually increases the dose; children one drop three times a day, gradually increased.

Dr. Kingsley reported a case of tetanus following a compound fracture of the forearm in a child seven years old. The fracture was reduced and the arm placed in a metallic splint. One week after the accident they sent for him, and he found the tongue swollen, with a few sores on it. The dressings on the arm were removed, but finding no swelling and the parts looking well, reapplied them, and at noon of the same day they sent for him again, when he found evidence of tetanus, and by evening tetanic convulsions were present.

Dr. Hadra saw the case also. Carbolic acid in conjunction with chloral and the bromides was used. By midnight the child was having violent spasms, and died in the third spasm. Muscular rigidity remained after death. Could any other procedure have saved the child?

After considerable discussion on the treatment of tetanus, Dr. Barnitz moved that Dr. Kingsley's treatment of this case, both medical and surgical (which had been described in detail), be endorsed by the association. The motion was unanimously carried.

The president appointed Dr. Bindley, Dr. Kingsley and Dr. Jackson to report cases or read papers at our next meeting.

Upon motion, the meeting adjourned.

JNO. S. LANKFORD, President.

WM. E. LUTER, Secretary.

